# Pilot Study on the Action of *Thymus vulgaris* Essential Oil in Treating the Most Common Bacterial Contaminants and *Salmonella enterica* subsp. *enterica* Serovar Derby in Poultry Litter

**DOI:** 10.3390/antibiotics12030436

**Published:** 2023-02-22

**Authors:** Michela Galgano, Francesco Pellegrini, Giuseppe Fracchiolla, Daniela Mrenoshki, Aya Attia Koraney Zarea, Angelica Bianco, Laura Del Sambro, Loredana Capozzi, Antonella Schiavone, Medhat S. Saleh, Michele Camero, Maria Tempesta, Francesco Cirone, Domenico Buonavoglia, Annamaria Pratelli, Alessio Buonavoglia

**Affiliations:** 1 Department of Veterinary Medicine, University Aldo Moro of Bari, Sp Casamassima Km 3, Valenzano, 70010 Bari, Italy; 2 Department of Pharmacy-Drug Sciences, University Aldo Moro of Bari, Via Orabona 4, 70125 Bari, Italy; 3 Department of Microbiology and Immunology, Veterinary Research Institute, National Research Centre (NRC), Cairo 12622, Egypt; 4 Istituto Zooprofilattico della Puglia e della Basilicata, Contrada San Pietro Piturno, Putignano, 70017 Bari, Italy; 5 Department of Animal Production, Faculty of Agriculture, Benha University, Banha 13736, Egypt; 6 Dental School, Department of Biomedical and Neuromotor Sciences, Via Zamboni 33, 40126 Bologna, Italy

**Keywords:** essential oils, poultry farms, *Escherichia coli*, *Mammaliicoccus lentus*, *Salmonella* Derby, antimicrobial activity

## Abstract

The indiscriminate use of antimicrobials in poultry farms is linked to the increase in multi-resistant bacteria. Accordingly, based on the antimicrobial properties of Thyme Essential Oil (TEO), the present study evaluated the effects of TEO on the reduction of common microbial contaminants and *Salmonella* on poultry litter. A litter bulk sample was collected in a broiler farm and qualitative/quantitative investigations identified *Escherichia coli* and *Mammaliicoccus lentus.* The experimental contamination with *Salmonella* Derby wild strain was also performed. All pathogens showed phenotypic and genotypic resistance to different classes of antibiotics. The litter, split in different units, was treated with aqueous solutions of TEO at different concentrations (5% to 1.25%), demonstrating its effectiveness in reducing the total number of bacteria. The strongest antibacterial action was observed at the lowest concentration against *Enterobacteriaceae*, with a growth reduction compared to the positive control of 73.3% and 77.8% against *E. coli* and *Salmonella* Derby, respectively, while towards *M*. *lentus* the reduction was 50%. Our data confirm the antimicrobial activity of TEO and suggest its possible application for the treatment of poultry litter as an effective and natural approach for the prevention of diseases caused by the most common bacteria that colonize poultry farms, counteracting the onset of antibiotic resistance.

## 1. Introduction

In recent years, the ever-increasing production of broilers and the related increase in the number and size of poultry farms has resulted in a significant environmental impact not only on the size of the farms but also on the production system adopted on the composition of the poultry diet and on the type of litter [1]. In addition to the environmental impact, the accumulation of microflora in the air, equipment, and litter of poultry farms is one of the most important factors negatively affecting the performance of industrial poultry [2]. Baykov and Stoyanov [3] demonstrated the presence of pathogens 3 km away from poultry farms. The Gram-positive bacteria that most commonly colonize poultry litter are cocci (*Staphylococcus* spp., *Streptococcus* spp., *Micrococcus* spp.) and bacilli (*Bacillus* spp.). The Gram-negative bacteria include that from the *Enterobacteriaceae* family, including *Escherichia coli* and *Salmonella* spp. [4], with the latter being able to survive in the intervals between production cycles, even after disinfection, thereby posing a serious health problem [5]. Without proper treatment, contaminating pathogens can spread to the external environment. Furthermore, considering that poultry litter represents a valuable resource, i.e., it can be used as fertilizer or as an energy source, it is necessary to take viable measures to minimize the health impact on poultry farms.

Also noteworthy is the problem of antimicrobial resistance (AMR), a growing threat to public health worldwide, and its impact on the prevention and treatment of infectious diseases. The first use of antibiotic drugs in poultry dates back to 1946 [6], and the first resistance was reported soon after in food animals by Starr and Reynolds [7]. Subsequently, several studies have demonstrated the link between the use of antibiotics at sub-therapeutic doses and the development of antimicrobial resistance in the microflora [8], and, although it is still a debated topic, the transport and the spread of microorganisms, antibiotics, and disinfectants in even distant areas can occur through the use of litter as fertilizer in the field, with a potentially negative impact on human health [9].

The broiler industry is now strongly geared towards eliminating the use of antibiotics throughout the broiler lifecycle, thus seeking innovative management systems [10]. In light of these considerations, the search for safe methods for poultry waste disinfection is a promising strategy, and one of the recent approaches for the control of bacterial populations during the production cycle is the inoculation of competitive bacterial populations or enzymes [11].

Even plant extracts, such as some essential oils (EOs), represent a valid alternative and, by virtue of their antimicrobial properties, find application in phytotherapy in the poultry field [12]. In vitro studies have shown that EOs counteract pathogenic bacteria, i.e., *E. coli*, *Salmonella enterica* (including serovars Enteritidis and Typhimurium), *Pseudomonas* spp., *Campylobacter* spp., *Staphylococcus* spp., *Streptococcus* spp., and fungal infections, i.e., *Microsporum* spp., *Trichophyton mentagrophytes*, *Malassezia* spp., and *Aspergillus* spp. [13]. Among them, thymus vulgaris L. essential oil (TEO) has high levels of antimicrobial activity and has often been compared with other oils in vitro [13] and in vivo [14].

Based on these observations, the aim of this work was to perform microbiological analyses of broiler litter at the end of the breeding cycle and to evaluate the bactericidal properties of TEO.

## 2. Results

### 2.1. Chemical Composition of TEO

The chemical composition of TEO was determined using GC/MS. About 25 components were identified and comprised 98.7% of the total detected constituents, as previously reported [15]. The major components were thymol (47%), p-cymene (19.6%), and γ-terpinene (9%), suggesting that the analyzed EO belongs to the thymol chemotype. A detailed description of TEO components is reported in Table S1 (Supplementary Files).

### 2.2. Bacteria Identification from Litter

Bacteriological screening of the bulk litter sample detected two species of bacteria with the researched phenotypic characteristics, mesophilic aerobic bacteria Gram-negative lactose-positive and Gram-positive mannitol salt-positive, identified as *E. coli* and *M. lentus*, respectively, while tests for *Salmonella* spp. before the experimental contamination were negative.

The multilocus sequence typing (MLST) analysis of the whole genome sequences of the isolated strains confirmed the identification of *E. coli* and *M. lentus* and the serotyping of *S.* Derby. The pubMLST tool assigned to the *M. lentus* the sequence type (ST), while for the *E. coli* isolate the type ST10 was predicted. Additionally, European Union Reference Laboratory-Verocytotoxin-producing *E. coli* (EURL-VTEC) predicted for the *E. coli* isolate the serotype O120:H3.

Similarly, the whole genome sequencing (WGS) analysis confirmed the characterization of the *S.* Derby strain used for the artificial contamination. Specifically, it was identified as *S.* Derby by SeqSero and rMLST, and pubMLST assigned the type ST682.

### 2.3. Screening for Multidrug Resistance (MDR) Activity

Details of the isolated strains about the phenotype and the genetic determinants of antimicrobial resistance are provided in Table 1.

Table 2 shows the resistance genes detected in the genome of individual bacteria and among these *E. coli* has the highest number of resistance genes. Among these resistance genes, the most noteworthy is the plasmid gene mph(C)_2, which encodes for active macrolides efflux, and which expresses phenotypic resistance to at least one of the four different classes of antibiotics, beta-lactams (OX, KZ), macrolides (E), glycopeptides (VA), and lincosamides (CD), a characteristic that makes it MDR. However, the same strain, despite the presence of multiple beta-lactam resistance genes, (Bla)AmpC1_Ecoli, (Bla) Penicillin_Binding_Protein_E. coli, and baeR, proved sensitive to multiple molecules belonging to the beta-lactam category, AMP, and different classes of cephalosporins, while it was also susceptible to AUG, allowing it to be considered an extended-spectrum beta-lactamase (ESBL) strain [16]. Similarly, the E. coli_mdf(A)_1gene coding for resistance to aminoglycosides and macrolides and to mdtO, which confers resistance to carbapenems and macrolides, were not phenotypically expressed. The antibiogram showed that *S*. Derby was resistant to AMP, OX, KZ, FOX, E, VA, CD, CS and IMI. The resistance to the beta-lactam antibiotics could be ascribed to the (Bla) TEM gene, encoding a beta-lactamase enzyme, while aac-(6′)1gene, associated with resistance to aminoglycoside resistance, was not phenotypically expressed, probably because it is not one of the most widely used antibiotics in poultry farms, so no environmental pressure triggers its expression [17]. On the other hand, the golS, mdsA and mdsB genes, which code for efflux pumps, explain resistance to other antibiotics. In *M. lentus*, the presence of the (Bla)Z_8 gene, a member of beta-lactamase, appears not to be linked to resistance to ampicillin but to other beta-lactam antibiotics, such as the oxacillin. It shows poor resistance with an intermediate sensitivity value when reading the antibiogram according to the Kirby Bauer method and also appears to be resistant to first-generation cephalosporins (KZ, FOX) and polymyxin E (CS), while regarding the presence of the mph(C)_2 gene, it cannot be associated with any resistance.

### 2.4. Antibacterial Activity of TEO

Preliminary investigation showed that the tested strains were highly sensitive to TEO in vitro, with total growth inhibition at the lowest dilution, 1.25% (*v*/*v*) corresponding to 112.5 µg/mL (*w*/*v*), with equal MIC and MBC values.

Total counts of mesophilic bacteria detected into plates before treatment, expressed as log_10_ Colony Forming Unit (CFU)/g of litter, are reported in Table 3. The mean count of detected mesophilic bacteria was 9.42 log_10_ CFU/g, and the total number of mesophilic bacteria in each plate was similar. The number of mannitol-positive *Staphylococcus* spp. was comparable to or slightly lower than the total mesophilic counts, approximately 9.00 log_10_ CFU/g. *Enterobacteriaceae* contamination levels were lower compared with the total mesophilic count, about 7.50 log_10_ CFU/g. The *Salmonella* count in the plates of the experimental Group B contaminated before treatment, as described in the experimental protocol, returned an average value of 9.00 log_10_ CFU/g.

Table 4 shows the mean rate of bacterial growth reduction, expressed as log_10_ CFU/g, in the experimental plate after 24 h and 48 h of treatment with TEO at different concentrations (*v*/*v*), and the relative percentage of growth reduction (%) compared to the positive control. The lowest growth reduction was observed by testing TEO at 1.25% with a reduction of 4.32 log_10_ CFU/g (45.85%) compared to the control, followed by a reduction of 6.2 log_10_ CFU/g and 7.28 log_10_ CFU/g (65.81% and 77.28%) in plates treated with 2.5% and 5% TEO, respectively. Mean total mesophilic count values performed 48 h after treatment with TEO showed no substantial changes in microbial growth compared to the 24 h evaluation.

The mean post-treatment growth reduction observed in both experimental Group A and Group B revealed that all strains were sensitive to the antimicrobial effect of TEO, showing a percentage reduction in growth rate ranging from 99.9% to 50% compared to the control, based on TEO concentration (Table 5). In the plate count of mannitol salt-positive staphylococci, a reduction of 7 log_10_ CFU/g compared to the control, corresponding to 77.7%, was observed after treatment with TEO at a concentration of 5%, while at the lower concentrations, 2.5% and 1.25%, a reduction of 6 log_10_ and 4.5log_10_, corresponding to 66.6% and 50%, respectively, was observed. No lactose-positive *Enterobacteriaceae* was observed at the highest concentration of TEO, suggesting a complete inhibition of bacterial growth. At the lower dilutions, 2.5% and 1.25%, a reduction in growth of 6.5 log_10_ CFU/g (86.6%) and 5.5 log_10_ CFU/g (73.3%), respectively, was observed compared to the control.

Treatment with TEO at 5% and 2.5% reduced *S.* Derby concentration to approximately 8 log_10_ CFU/g. A reduction of 7 log_10_ CFU/g was observed using a TEO concentration of 1.25%. These data, compared to the control, showed a range of growth reduction from 88.9% to 77.8%.

**Figure 1:**
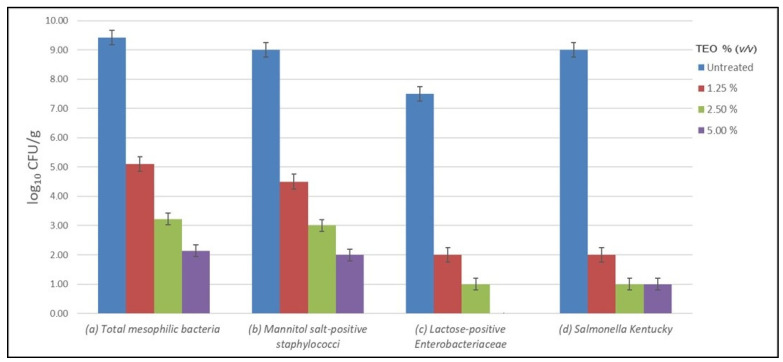
Bacterial growth of the tested strains in poultry litter treated with different concentration (*v*/*v*) of TEO after 24 h of incubation, compared to positive controls.

In Figure 1, the effect of different concentrations of TEO on total microbial growth and on individual strains compared to the control is reported. In the treated groups, the concentration of bacteria decreased proportionally with increasing TEO concentration. In particular, it was possible to observe a more marked reduction of *Enterobacteriaceae* and of lactose-positive *Enterobacteriaceae*, which at the highest concentration of TEO (5%) showed a total growth inhibition; on the other hand, at lower concentrations, a growth-inhibition effect is also observed, up to about one third of the number of colonies compared to those found in the untreated group. These latter data are comparable to those observed for *S.* Derby with all tested concentrations of TEO. For mannitol salt-positive Staphylococci, growth comparable to the total mesophilic one was observed after treatment, and the microbial load was halved compared to the control when the lowest concentration of TEO (1.25%) was used, and further decreased using the higher concentrations, never reaching total inhibition.

## 3. Discussion

There is strong evidence that EOs have a positive effect on the production performance of broilers, with no bacterial resistance reported so far [12]. Among all the components present in the EO of different *thymus* species, a pivotal role for antimicrobial activity is represented by the phenolic component, including carvacrol, thymol, and eugenol, whose presence defines the thymus EOs chemotype [18]. In addition, the antimicrobial activity of the phenolic component is higher when used in its entirety than when the main components are used individually, confirming the hypothesis of the synergistic effect [19]. However, it is not always easy to compare scientific data from different studies, as the percentage composition of EOs can be influenced by various factors, including geographic origin, climatic and soil conditions, growth cycle stage, and harvest seasons, which makes the chemical composition of EOs difficult to standardize [20]. Many studies were carried out in vitro to evaluate the effects of EOs on some Gram-positive and Gram-negative pathogenic bacteria as well as yeasts, such as *Candida albicans* and molds *Aspergillus niger*, and many of these studies tried to explain the mechanism of action of EOs against bacteria [21]. The complexity of the mechanisms of action is related to the chemical composition of EOs, which has a high diversity of molecules that act synergistically on a specific target [22].

This preliminary study aimed to investigate the effect of direct microemulsion of TEO at different concentrations on the growth of antibiotic-resistant bacteria in chicken litter. The values of total mesophilic bacteria and of the Gram-positive and Gram-negative ratio with a predominance of Gram-positive organisms that was detected in chicken litter was within the range reported in the literature, while the absence of *Salmonella* spp. highlighted the proper sanitary conditions of the litter of the tested farm [23].

The different resistance genes identified, phenotypically expressed or not, and the analysis of their sequences demonstrated the potential risk of the spread of multi-resistant clones in the indoor and outdoor environment of the poultry house. Among these, the most noteworthy one is the plasmid gene mph(A), which codes for the active efflux of macrolides, thought to be the most detected macrolide resistance gene in *E. coli* and the one most easily exchanged between bacteria with phylogenetically neighbor species, including *Salmonella* spp. [24]. Regarding beta-lactam resistance genes, the present investigation demonstrated that all the strains isolated from the litter showed resistance genes with different sensitivity to the tested antibiotics. Notably, all bacteria were resistant to cefazolin and only *S.* Derby was resistant to ampicillin, oxacillin, and cephalosporins of the first and second generation. Furthermore, several environmental and genetic factors may influence the phenotypic expression of antibiotic resistance genes/mutations and the ability to resist and spread worldwide [25]. Indeed, the mph(C)_2 gene detected in *M. lentus* cannot be associated with any resistance, since the expression of the gene alone seems insufficient to express drug resistance but only when associated with other genes, such as *MRS* (A) and *Erm*(Y) [26].

Resistance to antibiotics is extremely high in poultry litter due to the utilization of antibiotics used in the prevention of infectious disease [27]. Furthermore, it is possible to speculate that such resistance could result from use in the past of low-level, non-therapeutic, and antibiotic dietary supplements to stimulate growth, which may have contributed to the selection of antibiotic-resistant bacterial populations in the environment and in animals [8]. The prevalence rates of these genes are reaching alarming values. In staphylococci, the presence of (Bla)Z_8 gene ranged between 73–92% [28] and the prevalence of the mph(C) gene varied between 16.6% and 60% [29]. The prevalence of the resistant genes in *E. coli* are estimated to be 94.7% for (Bla)AmpC1_E.coli and only 6.7% for mtdO gene [30]. In *Salmonella* spp., the diffusion of (Bla) TEM gene ranged from 47.36% to 74.4% [31]. In addition, investigations conducted in natural and urban environments have disclosed a prevalence of 84.5% of the mdf(A)_1gene [32]. Since multidrug-resistant bacteria belonging to *Staphylococcaceae* and *Enterobacteriaceae* families are the cause of the main bacterial diseases in poultry, the reduction or at least the elimination of the use of antibiotics and the search for alternative molecules are a key step to facing and solving this problem [33]. One of the promising antibacterial preparations with a broad spectrum of action is EO from plants [13], and, consequently, one of the results of the present study showed that TEOs have strong antimicrobial activity against all tested strains, reducing the mean total counts of mesophilic bacteria, *Enterobacteriaceae* and *Staphylococcaceae*, compared to the control group. The action of TEO was more pronounced against *Enterobacteriaceae*, in particular against *E. coli*, which showed a reduction of 99.9% at the highest concentration (5%), while *S.* Derby and *M. lentus* at the same concentration showed a reduction of 88.9% and 77.7%, respectively. Previous studies demonstrated the activity of EOs on MDR in counteracting the main mechanisms of resistance: (i) inhibiting efflux [34], (ii) inhibiting the ESBL-mediated producer plasmidAmpC, TEM-1, TEM-2 or SHV-1 in gram-negative bacteria (ESBL) [35], and (iii) inhibiting the resistance genesmecA, mecR1, mecI, blaZ, blaR1, and blaI in the Gram-positive *Staphylococcus* epidermidis [36]. All these data confirm that OEs are a valid alternative to the use of antibiotics in farms without determining microbial resistance and without oil residues present in the final products [37].

At present, considering that chickens will become the most consumed meat globally even if they are a potential reservoir of zoonoses [38,39], the correct farm management is a priority for health in the perspective of One Health. The World Health Organization, in its recent report on antimicrobial resistance, outlined a global plan including the integrated surveillance of food-producing animals and the food chain [40], considering that the poultry environment can be seen as a worrying source of multidrug-resistant bacteria.

Despite many gaps that are still to be filled (i.e., to standardize a suitable and effective protocol that can be used within clinical trials, to determine the optimal dosage and how long its effectiveness lasts, and to understand the mechanism of action of the different components) and the fact that further analysis would be needed to rule out any methodological flaws (i.e., an insufficient number of replicates, different environmental conditions, or a short application time), we can still assert that bioactive agents such as EOs have multiple potential applications. In conclusion, the results of the present study confirm the antimicrobial activity of TEO against the most common bacteria that colonize poultry farms, i.e., *E. coli*, *M. lentus* and *S*. Derby. Therefore, TEO is a natural bioactive agent candidate for the treatment of poultry litter, and its use should be strengthened to counteract the onset of antibiotic resistance while safeguarding broilers health and environmental hygiene.

## 4. Materials and Methods

### 4.1. Litter Bulk Sample and Bacteria Identification

The experiment was performed on a litter bulk sample (approximately 3.5 kg) of broiler poultry, harvested with a sterile spatula at the end of the production cycle, from five different areas in a poultry house of around 20,000 free-range broilers on a semi-intensive poultry farm in the province of Foggia in southern Italy.

The collected bulk sample was mixed, and quantitative and qualitative bacterial contamination was evaluated before the treatment with the TEO aqueous solutions. The number of bacteria, expressed in CFU/g of litter, was evaluated in accordance with standard PN-EN ISO 18593:2005, and bacterial count assays were performed according to PN-EN ISO 21528–2:2005. Quantitative analyses of mesophilic aero-bacteria, lactose-positive *Enterobacteriaceae*, and mannitol salt-positive staphylococci were determined by selective and differential media (MacConkey Agar and Mannitol Salt Agar, Oxoid, Milan, Italy) [41]. In addition, pre-enrichment and PN-EN ISO 6579:2002 methods were employed for *Salmonella* spp. Isolation, and bacterial count assays were performed as described by Shanmugasamy et al. [42]. The same bacterial counts were also determined after EO treatments.

### 4.2. Antimicrobial Susceptibility

The antimicrobial susceptibility of the isolated strains was determined by the disk diffusion method according to the CLSI standard. Disks containing ampicillin (AMP, 10 µg), amoxicillin + clavulanic acid (AUG, 30 µg), oxacillin (OX, 1 µg), cefazolin (KZ, 30 µg), cefoxitm (FOX, 30 µg), ceftriaxone (CRO,30 µg), ceftaxime (CTX, 30 µg), cefuroxime (CXM, 30 µg), erythromycin (E, 15 µg), doxycycline (DO; 30µg), tetracyclin (TE, 30 µg), gentamicin (CN, 10 µg), amikacina (AK, 30 µg), vancomycin (VA, 30 µg), clindamycin (CD, 2 µg), co-trimoxazole(SXT, 25 µg), colistin sulphate (CS, 10 µg), and imipenem (IMI, 10 µg) were used. The antibiotics were selected according to the standardized therapeutic protocols available for infections by Gram-negative and Gram-positive bacteria according to Clinical & Laboratory Standards Institute (CLSI) guidelines and data in reviewed literature, which include the most common antibiotics used in the poultry industry. *E. coli* ATCC 25922 and *S. aureus* ATCC 11622 were used for quality control.

### 4.3. Whole-Genome Sequencing and Strains Typing

Genomic DNA was extracted from single colonies isolated in TSA (Trypticase Soy Agar, Oxoid, Milan, Italy), using DNeasy Blood and Tissue Kit (Qiagen, Hilden, Germany), according to the manufacturer protocol. The final DNA concentration was estimated by Qubit Fluorometer using Qubit dsDNA HS Assay (Thermo Fisher Scientific). For each isolate, a paired-end genomic library was prepared using the Nextera DNA Flex Library preparation kit (Illumina, San Diego, CA, USA). Sequencing was performed using MiSeq Reagent Kitv 2 (2250 bp) on Illumina MiSeq platform (Illumina, San Diego, CA, USA). Raw sequence reads (FASTQ dataset) from Illumina sequencing were trimmed (Trimmomatic [43]—GalaxyVersion 0.36.6) for the removal of adaptor sequences and quality control purposes, and de novo were assembled (SPAdes 3.12.0) [44] using the European Galaxy server (https://usegalaxy.eu/, accessed on: 25 July 2022). For species identification, FASTA files of the assemblies were uploaded onto the rMLST online free database (https://pubmlst.org/rmlst/, accessed: 25 July 2022). The European Union Reference Laboratory (EURL) for *E. coli* (EURL-VTEC) and SeqSero version 1.2, which are available at the Center for Genomic Epidemiology (http://www.genomicepidemiology.org/, accessed: 25 July 2022), were used to predict the serotype of *E. coli* and *S. enterica* isolates, respectively. Moreover, with the aim of identifying the antibiotic resistance genes and plasmids, the draft genome of each strain was analyzed using the software ABRicate (Galaxy Version0.8), which includes different preloaded databases [ARG-ANNOT (Guptaetal., 2014), NCBI AMR Finder Plus [45], CARD [46], ResFinder [47] and Plasmid Finder [48].

### 4.4. EO: Compound Identification and Dilution Design

The choice of TEO and the concentration to be used was based on its antimicrobial effect reported in the literature [49]. Commercially available natural TEO (Specchiasol S.r.l., Bussolengo, VR, Italy) stored in a brown glass bottle at the temperature of 0–4 °C was used in the experimental design. The composition of TEO was confirmed by chromatography hyphenated with mass (GC/MS) technique [15,49]. To achieve the proper solubilization of TEO, suitable for all the experimental procedures, the mother solution was serially diluted in 5mL Brain Heart Infusion (BHI) (Oxoid, Milan, Italy) with 10% dimethyl sulfoxide (DMSO), obtaining solutions ranging from a concentration of 5% (*v*/*v*) to 1.25% (*v*/*v*), corresponding to 450–112.5 µg/mL. The emulsion was then sonicated for 30 min and vortexed for 8 min to obtain a stable oil emulsion.

### 4.5. Experimental Design

After microbiological screening, given the absence of *Salmonella* spp. and to evaluate the antimicrobial effect of TEO on all bacteria of our interest, the litter bulk sample was split in eight units of 110 g each and distributed into seven different sterile Petri dishes (140 mm), generating three study groups:

- Experimental Group A: three litter samples treated with 0.2mL TEO 5%, 2.5%, and 1.25% (*v*/*v*), respectively.
- Experimental Group B: three litter samples artificially contaminated by spraying 0.2 mL of a 10^8^ CFU/mL suspension of *S. enterica* subsp. *enterica* ser. Derby (hereafter, *S.* Derby) and after 12h treated with 0.2mL TEO 5%, 2.5%, and 1.25% (*v*/*v*), respectively.
- Control Group: one litter sample for each group (A and B), sprayed with 0.2 mL of pure water.

After treatment, the samples were maintained at 24–26 °C for the whole duration of the test. The total bacterial count was determined in litters of Group A and Group B at T0 (before TEO treatment) and at 24 h and 48 h after TEO treatment. Similarly, the bacterial count for Lactose-positive *Enterobacteriaceae* and Mannitol salt-positive *Staphylococcaceae* as well as the bacterial count for contaminant *S.* Derby were assessed at T0 and at 24 h after TEO treatment. Each assay was performed in triplicate, and the values of growth reduction were expressed as log-reduction and as a percentage in the treated group compared to the control group with its standard deviation. Data are reported as means of log_10_ viable cell densities ±standard deviation of log densities.

### 4.6. Antibacterial Activity of TEO

Minimal inhibitory concentration (MIC) and minimum bactericidal concentration (MBC) in broth dilution assay were assessed to investigate the potential antibacterial activity of TEO against *Enterobacteriaceae* pathogens, *E. coli* (ATCC 25922) and *S.* Derby (wild strain), and against mannitol salt-positive staphylococci, *S. aureus* isolates (ATCC 11622) according to CLSI [49,50]. These strains were part of the culture collections of the Department of Veterinary Medicine of the University of Bari “Aldo Moro”.

## Figures and Tables

**Table 1:** Antimicrobial resistance profiles of strains isolated from poultry litter.

Drugs	Bacterial Strains		
	*M. lentus*	*E. coli*	*S.* Derby
AMP	S	S	R
AUG	S	S	S
OX	I	R	R
KZ	R	R	R
FOX	R	I	R
CXM	I	S	I
CRO	S	S	S
CTX	S	S	S
E	S	R	R
AK	S	S	S
TE	S	S	S
DO	S	S	S
CN	S	S	S
VA	S	R	R
CD	S	R	R
SXT	S	S	S
CS	R	I	R
IMI	S	I	R

AMP: ampicillin; AUG: Amoxicillin/clavulanic acid; OX: oxacillin; KZ: ceftazolin; FOX: cefoxitin; CXM: cefuroxime; CRO: ceftriaxone; CTX: cefotaxime; E: erythromycin; AK: amikacin; TE: tetracycline; DO: doxycycline; CN: gentamicin; VA: vancomycin; CD: clindamycin; SXT: co-trimoxazole; CS: colistin sulfate; IMI: imipenem. S: sensitive; I: intermediate; R: resistant.

**Table 2:** Strains identified with combinations of phenotypic ^1^ and genetic resistance ^2^ to different antibiotics.

Detected Bacteria	Resistance Phenotype	Detected Resistance Genes and Mutations (n)
*M. lentus*	*KZ*; *FOX*; *CS*	*blaZ_8*; *mph(C)_2*
*E. coli*	*OX*; *KZ*; *E*; *VA*; *CD*	*(Bla)AmpC1_E. coli*; *(Bla)Penicillin_Binding_Protein_Ecoli*; *baeR*; *(Phe)CatB4*; *mdtO*; *evgA*; *Ecoli_mdfA*; *mdtG*; *mdtM*; *emrA*; *emrB*; *Ecoli_acrA*; *mdtF*
*S.* Derby	*AMP*; *OX*; *KZ*; *FOX*; *E*; *VA*; *CD*; *CS*; *IMI*	*(Bla)TEM-150*; *aac-(6′)1*; *golS*; *mdsA*; *mdsB*

^1^ Phenotypic resistance to antibiotics: AMP: ampicillin; OX: oxacillin; KZ: cefazolin; CXM: cefuroxime; FOX: cefoxitin; E: erythromycin; VA: vancomycin; CD: clindamycin; CS: colistin-sulphate; IMI: imipenem; ^2^ Category of antibiotics and relative antibiotics resistance genes detected: β-lactam resistance genes: *blaZ_8*; *(Bla)AmpC1_E.coli*; *(Bla)Penicillin_Binding_Protein_E.coli*; *baeR*; *(Bla)TEM-150*; Macrolide resistance gene: *mph(C)_2*; *(Phe)CatB4*; *mdtO*; *evgA*; Carbapenem resistance genes: *mdtO*; Aminoglycosides resistance gene: *E.coli_mdfA*; Quinolone resistance gene: *aac-(6′)1*.; Multidrug resistance protein: *mdtG*; *mdtM*.; Efflux pumps genes: *emrA*; *emrB*; *E.coli_acrA*; *mdtF*; *golS*; *mdsA*; *mdsB*.

**Table 3:** Screening and count of total mesophilic bacteria expressed as log_10_ CFU/g detected in broiler litter before treatment with TEO.

Bacterial Strains	T0
Total mesophilic count	9.42
Mannitolsalt-positive *Staphylococcaceae* (*M. lentus)*	9.00
Lactose-positive *Enterobacteriaceae* (*E. coli*)	7.50
*Salmonella enterica* ser. Derby (experimental infection)	9.00

T0: bacterial count expressed as log_10_ CFU/g in litter before treatment with TEO.

**Table 4:** Total mesophilic bacteria, expressed as log_10_ CFU/g, in broiler litter before (T0) and 24 h and 48 h after treatment with TEO at different concentrations (5%, 2.5%, 1.25%), with relative percentage reduction in growth (%) compared to the positive control.

T0	TEO	24 h		48 h	
		log_10_ CFU	Reduction %	log_10_ CFU	Reduction %
9.42	5%	7.28	77.28%	7.28	77.28%
	2.5%	6.2	65.81%	6.2	65.81%
	1.25%	4.32	45.85%	3.89	41.29%

T0: bacterial count expressed as (log_10_ CFU/g) in litter before treatment with TEO; TEO: concentration % (*v*/*v*); reduction %: growth reduction expressed as a percentage in the treated group compared to the control group.

**Table 5:** Microbial growth of each strain expressed as log_10_ CFU/g, and percent reduction in growth (%) compared to the positive control after 24 h treatment with TEO at different concentrations.

TEO	Mannitol Salt-Positive *Staphylococcaceae*		Lactose-Positive *Enterobacteriaceae*		*Salmonella* Derby	
	log_10_ CFU/g	Reduction%	log_10_ CFU/g	Reduction %	log_10_ FCU/g	Reduction%
5%	7.00	77.7	7.5	99.9	8.00	88.9
2.5%	6.00	66.6	6.5	86.6	8.00	88.9
1.25%	4.50	50	5.5	73.3	7.00	77.8

TEO: concentration % (*v/v*); reduction %: growth reduction expressed as a percentage in the treated group compared to the control group.

## Data Availability

Not applicable to this article.

## Supplementary Materials

The following supporting information can be downloaded at: https://www.mdpi.com/article/10.3390/antibiotics12030436/s1. Table S1: Chemical composition of *Thymus vulgaris* Essential Oil. Refs. [51,52,53,54] are cited in Supplementary Materials.
