# Supplementary material for: Pilot Study on the Action of Thymus vulgaris Essential Oil in Treating the Most Common Bacterial Contaminants and Salmonella enterica subsp. enterica Serovar Derby in Poultry Litter
Source: Antibiotics (Basel). 2023 Feb 22;12(3):436. doi: 10.3390/antibiotics12030436 (PMC10044251; doi:10.3390/antibiotics12030436)
Supplement: Supplementary file 1 [file antibiotics-12-00436-s001.zip › antibiotics-2210348-supplementary.pdf]

antibiotics

Supplementary Files

The pure EO of *Thymus Vulgaris* (TEO), was provided by Specchiasol S.r.l. (Bussolengo, VR, Italy) and were stored in a brown glass bottle at a temperature of 0–4°C. Solvents (analytical grade), *n*-alkanes standard mixture C10–C40, and all standard compounds were purchased from Supelco Sigma-Aldrich S.r.l. (Milano, Italy). Filters were supplied by Agilent Technologies Italia S.p.a (Milano, Italy).

Gas Chromatography/Mass Spectrophotometry (GC/MS)

The gas chromatographic analyses of the EOs were performed on an Agilent 6890 N gas chromatograph equipped with a 5973 N mass spectrometer, provided with a HP-5 MS (5% phenylmethylpolysiloxane, 30m, 0.25mm i.d., 0.1µm film thickness; J & W Scientific, Folsom) capillary column. The following temperature programmer was used: 5 min at 60°C, then 4°C/min to 220°C, then 11°C/min to 280°C, hold for 15min, for a total run of 65min. The injector and detector temperatures were 280 °C; the carrier gas was He; the flow rate was 1mL/min; the split ratio was 1:50; the acquisition range was 29–400m/z in electron-impact (EI) mode; and the ionization voltage was 70 eV [51].

Compound Identification

For chemical characterization, the EOs were diluted 1:100 in ethyl acetate and after filtration, 1µL of each EO solution was injected into the GC-MS. Qualitative analyses were carried out by comparing the calculated linear retention indices (LRIs) and similarity index of mass spectra (SI/MS) for the obtained peaks with the arithmetic index (AI) and the analogous data reported in the literature and in the NIST 2017 databases (NIST 17, 2017), respectively. The LRI of each compound was determined using temperature programming analysis and was calculated using the Vandendool and Kratz equation [52] related to a homologous series of n-alkanes (C10–C40) under the same operating conditions. The SI/MS were determined as reported by Koo et al. [53].

The component relative percentages were calculated based on GC peak areas without using correction factors.

Table S1. Chemical composition of *Thymus vulgaris* Essential Oil.

N	Components	LRI	AI	*Thymus vulgaris*	
				**Area±SEM**	**SI/MS**
1	Ethyl propanoate	714	714	0.1±0.01	86
3	cyclene	920	919	0.13±0.090	94
4	α-pinene [a]	930	931	1.81±0.10	95
5	camphene [a]	952	952	1.89±0.11	96
6	β-thujene	968	968	0.71±0.06	93
7	mushroom alcohol	975	975	0.4±0.02	83
8	β-pinene[a]	982	980	0.56±0.03	94
9	β-myrcene	990	991	1.4±0.08	86
10	α-phellandrene[a]	1002	1003	0.15±0.01	91
11	o-cymene	1021	1021	19.6±1.5	95
12	eucalyptol [a]	1023	1023	0.9±0.05	99
13	limonene[a]	1030	1032	0.6±0.04	91
14	γ-terpinene[a]	1062	1064	9±1	94
15	terpinolene	1083	1085	1.3±0.9	97
16	β-linalool[a]	1100	1101	4±1	97
17	camphor	1145	1146	1.7±0.8	98
18	terpineol, cis-β-	1147	1147	0.13±0.010	90
19	endo-borneol	1166	1167	1.8±0.7	97
20	terpinen-4-ol	1171	1171	1.8±0.8	96
21	α-terpineol	1178	1179	0.12±0.010	86
22	anisole	1234	1235	0.4±0.06	90
23	anisole, 2-isopropyl-4-methyl-	1244	1244	0.42±0.050	94
24	thymol[a]	1290	1290	47±3	94
25	caryophyllene[a]	1415	1415	2.2±0.9	99
26	caryophylleneoxyde	1596	1592	0.6±0.03	91
	% Characterized	/	/	98.7	/
	Others	/	/	1.30	/

[a]: standard compounds. Linear retention index (LRI) on HP-5MS column was experimentally determined using a homologous series of C10-C40 alkanes standard mixture [52]. Arithmetic index (AI) was taken from Adams [54] and/or the NIST 2017 Database (NIST 17, 2017. Mass Spectral Library (NIST/EPA/NIH). Gaithersburg, USA: National Institute of Standards and Technology. Last access 12_2021). Similarity index/mass spectrum (SI/MS) was compared with data reported on NIST 2017 Database and were determined as reported by Koo et al. [53]. Relative percentage values are means of three determinations with a structural equation modeling (SEM) in all cases below 10%.
